# Reexpansion Pulmonary Edema following Laparoscopy-Assisted Distal Gastrectomy for a Patient with Early Gastric Cancer: A Case Report

**DOI:** 10.1155/2012/863163

**Published:** 2012-11-28

**Authors:** Kazuhito Yajima, Tatsuo Kanda, Ryo Tanaka, Yu Sato, Takashi Ishikawa, Shin-ichi Kosugi, Tadayuki Honda, Katsuyoshi Hatakeyama

**Affiliations:** ^1^Division of Digestive and General Surgery, Niigata University Graduate School of Medical and Dental Sciences, 1-757 Asahimachi-dori, Niigata 951-8510, Japan; ^2^Advanced Disaster Medical and Emergency Critical Care Center, Niigata University Medical and Dental Hospital, 1-754 Asahimachi-dori, Niigata 951-8520, Japan

## Abstract

We report here a case of reexpansion pulmonary edema following laparoscopy-assisted distal gastrectomy (LADG) for early gastric cancer. A 57-year-old Japanese woman with no preoperative comorbidity was diagnosed with early gastric cancer. The patient underwent LADG using the pneumoperitoneum method. During surgery, the patient was unintentionally subjected to single-lung ventilation for approximately 247 minutes due to intratracheal tube dislocation. One hour after surgery, she developed severe dyspnea and produced a large amount of pink frothy sputum. Chest radiography results showed diffuse ground-glass attenuation and alveolar consolidation in both lungs without cardiomegaly. A diagnosis of pulmonary edema was made, and the patient was immediately intubated and received ventilatory support with high positive end-expiratory pressure. The patient gradually recovered and was weaned from the ventilatory support on the third postoperative day. This case shows that single-lung ventilation may be a risk factor for reexpansion pulmonary edema during laparoscopic surgery with pneumoperitoneum.

## 1. Introduction 

Due to advances in instruments and surgical techniques, laparoscopic surgery has been widely used in recent years for the treatment of early gastric cancer [1]. The many advantages of laparoscopic gastrectomy, including reduced surgical invasiveness, less postoperative pain, better cosmetic outcomes, and faster recovery after surgery, are well documented [2, 3]. Although surgical stress and tissue damage are minimized by laparoscopic techniques, laparoscopic surgery is associated with the risk of serious adverse events that are laparoscopic specific. These complications are mainly a result of prolonged pneumoperitoneum with concomitant high intraabdominal pressure. Reexpansion pulmonary edema (RPE) is a potentially life-threatening complication. Morbidity is caused by the rapid reexpansion of collapsed lungs, a process associated with the treatment of pleural effusion, pneumothorax, and single-lung ventilation. We herein report a case of reexpansion pulmonary edema following laparoscopy-assisted distal gastrectomy (LADG) associated with unintended single-lung ventilation.

## 2. Case Report 

A 57-year-old Japanese woman (body height: 146 cm; body weight: 54.3 kg; body mass index: 25.3 kg/m^2^) was diagnosed with early adenocarcinoma of the middle third of the stomach. She had no history of smoking, lung disease, or heart disease. Preoperative laboratory data were normal. Respiratory function tests showed that her vital capacity was 2160 mL, and forced expiratory volume in one second was 1640 mL. Chest radiography did not reveal any notable findings. Blood gas analysis (BGA) was not performed preoperatively. 

**Figure 1:**
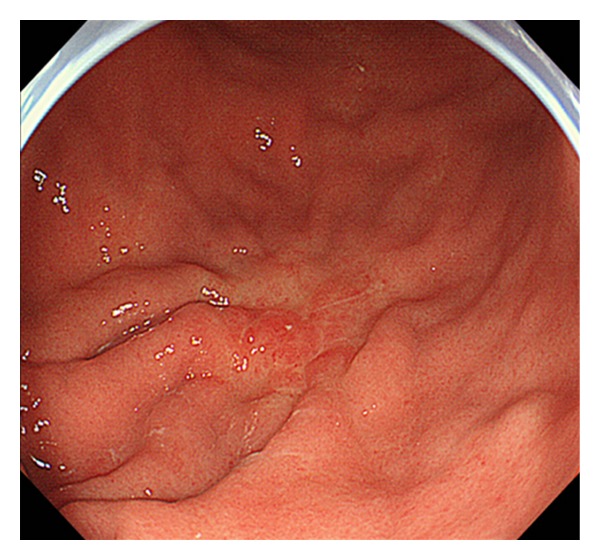
Gastrointestinal endoscopy revealed a depressed-type tumor in the greater curvature of the middle third of the stomach. Biopsy specimens showed a moderately to poorly differentiated adenocarcinoma of the stomach.

Upper gastrointestinal endoscopy revealed a depressed-type tumor in the greater curvature of the middle third of the stomach. The tumor was classified as a moderately to poorly differentiated adenocarcinoma by biopsy. Endoscopically, the tumor invasion was evaluated as not reaching the submucosa, but the tumor had a concomitant peptic ulcer scar (Figure 1). Accordingly, distal gastrectomy using a laparoscopic approach was recommended for this early gastric cancer (cT1N0M0, stage IA).

**Figure 2:**
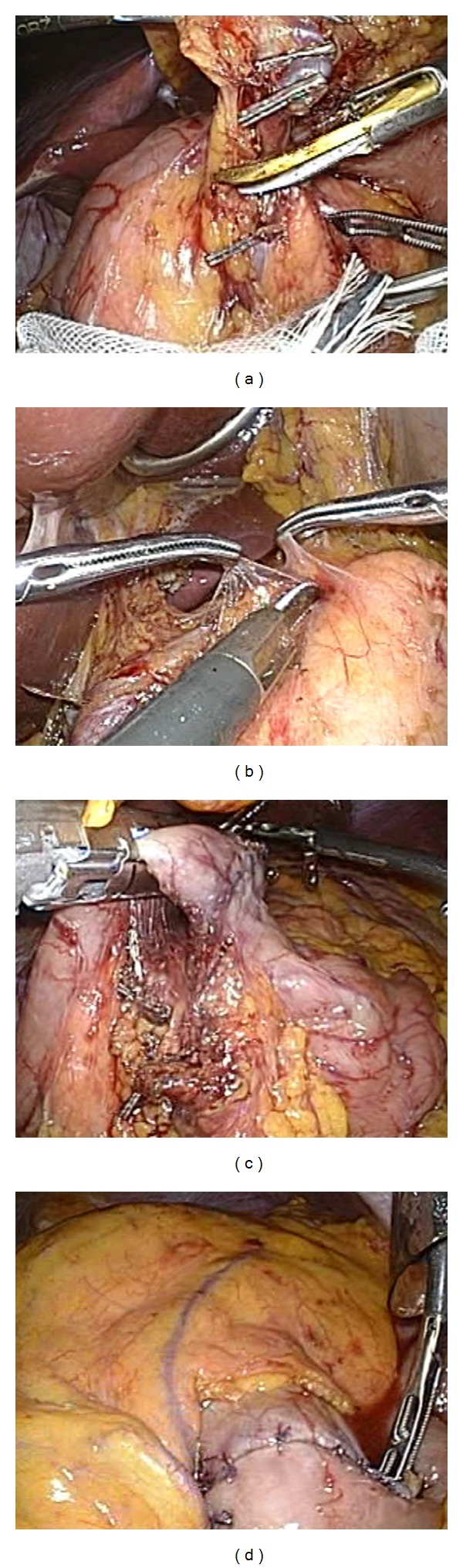
Intraabdominal findings from the laparoscopy-assisted distal gastrectomy with lymphadenectomy. (a) Dissection of the infrapyloric lymph nodes (station 6) from the pancreatic head: the right gastroepiploic vessels were exposed and divided. (b) Dissection of lymph node stations 7, 8a, 9, and 11p: suprapancreatic lymph nodes and lymph nodes around the celiac axis were dissected along the common hepatic artery and the splenic artery. (c) Transection of the duodenum: the duodenum was cut 1 cm distal to the pylorus using an endoscopic stapling device (Endo GIA, Duet TRS, Covidien, Tokyo, Japan). (d) Anastomosis: a Roux-en Y gastrojejunostomy was made. The jejunal limb was pulled up through the retrocolic route.

The LADG procedure in the present case was carried out as follows: the patient was positioned in the supine position with the legs apart and head-up tilt. A pneumoperitoneum was created using carbon dioxide via a Veress needle, and the maximum pneumoperitoneum pressure was set at 10 mmHg. Distal gastrectomy was completed with laparoscopic manipulations through five trocars, and a D1 lymphadenectomy with dissection of stations 8a, 9, and 11p [4] was also performed. The resected stomach was removed from a 5 cm minilaparotomy placed in the upper middle abdomen, and a gastrojejunostomy was made extracorporeally using the Roux-en-Y procedure. Intraoperative findings are shown in Figure 2. The total operative time and the duration of pneumoperitoneum were 309 minutes and 214 minutes, respectively. The blood loss was less than 10 mL. 

General anesthesia was induced using propofol (1% Diprivan injection, AstraZeneca Co., Osaka, Japan) and rocuronium bromide (Eslax Intravenous, MSD K.K., Tokyo, Japan). Remifentanil hydrochloride (Ultiva, Janssen Pharmaceutical K.K., Tokyo, Japan) was also administered. An epidural anesthesia using ropivacaine hydrochloride hydrate (Anapeine injection, AstraZeneca Co., Osaka, Japan) was also administered. The intratracheal tube (7.0 mm ID) was inserted transorally and placed 21 cm from the incisors and inflated with 4 mL of cuff air. Upon noticing a decrease in the monitored SpO_2_ levels, the intratracheal tube was pulled back 1 cm under bronchofiberscopic observation 247 minutes after the start of anesthesia. The results of BGA during anesthesia and the postoperative course are shown in Table 1. 

**Figure 3:**
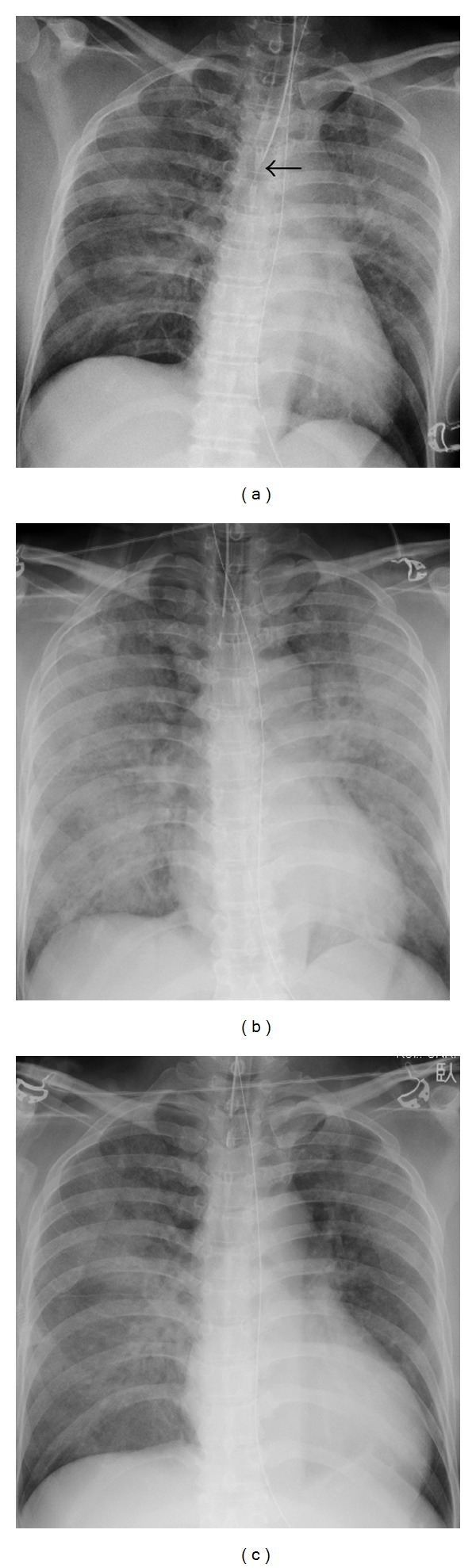
(a) A postoperative chest radiograph taken in the operating room showed bilateral diffuse ground glass attenuation. The central shadow was not widened: the cardiopulmonary rate was 48%. The tip of the intratracheal tube was located near the tracheal bifurcation (black arrow). (b) A chest radiograph demonstrated progression of the diffuse ground glass attenuation and appearance of alveolar consolidation. The photograph was taken in the intensive care unit 2 hours after surgery. (c) A chest radiograph revealed significant resolution of pulmonary abnormalities 3 days after the operation.

The total time under anesthesia was 409 minutes. The total administered fluid intake was 2560 mL, and urine output during surgery was 330 mL. Blood pressure and heart rate remained stable throughout the surgery.  Figure 3(a) shows the chest radiograph that was taken in the operating room just after surgery was completed. 

The patient was extubated in the operating room and returned to the surgical ward as her respiratory condition was regarded as acceptable. One hour after surgery, the patient complained of dyspnea and rapidly developed respiratory failure: pulse oximetry revealed that the blood oxygen saturation decreased to 85% despite the use of an oxygen mask (10 L/min). Arterial BGA indicated the following results: pH 7.237, pO_2_ 56.2 mmHg, and pCO_2_ 63.9 mmHg. A large amount of pink frothy sputum was discharged from the airway and nasogastric tube. A chest radiograph demonstrated progression of diffuse ground glass attenuation and the appearance of alveolar consolidation (Figure 3(b)). On the basis of these findings, a diagnosis of pulmonary edema was made. 

The patient was immediately intubated and received ventilatory support using the Puritan Bennett 840 Ventilator System (Covidien, Tokyo, Japan), set on the synchronized intermittent mandatory ventilation plus pressure support (PS) mode, with a tidal volume of 450 mL, frequency of 20 breaths/minutes, positive end-expiratory pressure (PEEP) of 10 mmHg, PS of 8 mmHg, and FiO_2_ of 100%, in the intensive care unit. A dose of 500 mg of methylprednisolone sodium succinate (Solu-Medrol, Pfizer Japan, Tokyo, Japan) was administered by intravenous bolus, and sivelestat sodium hydrate (Elaspol, Ono Pharmaceutical Co., Ltd, Osaka, Japan), a selective inhibitor of neutrophil elastase, was also administered (0.23 mg/kg/hr) for three days. Fiber optic bronchoscopy revealed that the frothy secretions originated from both lungs.

The patient's respiratory condition improved gradually, and she was extubated on the third postoperative day (POD) (Figure 3(c)). Thereafter, the patient recovered uneventfully. She started a diet on the fifth POD and was discharged on the 15th POD. 

## 3. Discussion

We have described a 57-year-old woman who developed severe bilateral pulmonary edema following LADG for early gastric cancer. To characterize this rare but life-threatening disease, we searched the PUBMED and Japana Centra Revuo Medicina (Vor.5) databases using the keywords “laparoscopy” and “pulmonary edema.” As of October 2011, there were only nine case reports including reference lists describing pulmonary edema following laparoscopic surgery. The nine published cases and the current case are summarized in Table 2 [5–12]. Four cases were from Japan [5, 6, 9, 10], three from South Korea [7, 11, 12], and the remaining two from the United States [8].

Of the 10 cases with pulmonary edema following laparoscopic surgery (Table 2), five patients were men and five were women with a median age of 44.5 years (range: 23–73 years). Three patients had preoperative comorbidity: however, only one patient had preoperative cardiopulmonary comorbidities (Case  7). Three patients had a malignant disease, which included cecal cancer, prostate cancer, and gastric cancer. In three patients, pulmonary edema was associated with accidental single-lung ventilation during surgery. The median operative time was 166 minutes (range: 50–330 minutes), and median infusion during the surgery was 2225 mL (range: 1750–8000 mL). The pulmonary edema was unilateral in five patients and bilateral in five patients. 

Common causes of pulmonary edema include heart failure with left ventricular dysfunction, fluid overload, and renal failure. Morrisroe et al. [8] reported two cases of pulmonary edema following laparoscopic living-donor nephrectomy. The infusion volumes during surgery for these two nephrectomy cases were 7700 mL in 5 hours and 8000 mL in 5.5 hours, respectively. The authors presumed that the infusion overload may have been the main cause of the postoperative pulmonary edema. Patient position during an operation is also an important issue to consider when determining the association between volume overload and perioperative pulmonary edema. Several reports suggested that a steep Trendelenburg position could be one of the risks for perioperative pulmonary edema [5, 11–13]. Stoelting [13] reported a case of severe pulmonary edema following total pelvic exenteration in a 30-year-old woman with alveolar rhabdomyosarcoma of the pelvis. Stoelting [13] presumed that the steep Trendelenburg position was a possible cause: the steep position led to further elevation of the high central venous pressure thereby provoking the development of pulmonary edema. 

In the present case, the chest radiograph taken at the end of the operation did not show cardiomegaly, and the infused volume for this patient (2560 mL lactated Ringer's solution in 5 hours) did not appear to be an overload. Moreover, the patient was positioned with a head-up tilt during the laparoscopic surgery. Cardiac failure or fluid overload was unlikely to account for perioperative pulmonary edema in the present case.

RPE is a particular form of pulmonary edema. In general, RPE is well known as a complication associated with treatment for pleural effusion and pneumothorax [14]. The reported incidence rate of RPE following spontaneous pneumothorax ranges from 0.9% to 14% [15, 16]. The clinical presentations of RPE are rapid onset of dyspnea and/or tachypnea. Pink frothy sputum is one of the important signs used to make a clinical diagnosis. Mahfood et al. [17] reviewed 47 cases of RPE reported between 1958 and 1987. Based on their study, 64% of the patients developed RPE within one hour of lung reexpansion, and the remainder developed it within 24 hours. Interestingly, RPE could occur not only in the collapsed lung but also in the contralateral lung or in both lungs. It is noteworthy that the study indicated that middle-aged women were more likely to be affected by RPE: the cohort was composed of 9 men and 38 women with an average age of 42 years.

RPE associated with surgery can occur after single-lung ventilation, although the exact pathophysiology is still unknown. Many cases of RPE following single-lung ventilation occurred in patients undergoing thoracoscopic surgery, which requires intentional single-lung ventilation [18–21]. Hong et al. [7] reported a case of RPE that occurred in a patient with a body mass index of 38.6 kg/m^2^ who underwent laparoscopic gastric banding. In that case, single-lung ventilation accidentally occurred during surgery and continued for approximately 50 minutes. Cephalad movement of the carina during laparoscopic surgery was confirmed to cause this and may have been associated with high insufflation pressure [22]. In the present case, the results of BGA worsened with time during surgery after initiation of pneumoperitoneum. Moreover, a chest radiograph indicated that the top of the intubation tube was positioned just above the tracheal bifurcation even though the tube was relocated during surgery. These findings suggested that unintended single-lung ventilation, which might be caused by upward-migration of the diaphragm associated with pneumoperitoneum, triggered RPE in the present case.

Carbon dioxide (CO_2_) is generally used for pneumoperitoneum because it is quickly absorbed from the peritoneal cavity into the circulation. However, the absorbed CO_2_ might induce hemodynamic, pulmonary, renal, splanchnic, and endocrine pathophysiological changes [23]. Pulmonary complications of laparoscopic surgery with CO_2_ pneumoperitoneum are represented by hypercapnia, hypoxemia, acidosis, barotrauma, pulmonary edema, atelectasis, gas embolism, and pneumothorax. Karapolat et al. [24] demonstrated histologically that CO_2_ pneumoperitoneum caused oxidative stress injury to lung tissue including intra-alveolar hemorrhage, congestion, and leukocyte infiltration in a rodent model. However, at present there is no clinical evidence indicating that CO_2_ pneumoperitoneum is a risk for pulmonary edema. The clinical significance of hypercapnia associated with pneumoperitoneum is more important, because an increasing number of cancer surgeries are being performed using a laparoscopic approach, a process that requires prolonged pneumoperitoneum and has an increased risk for hypercapnia.

The treatment for pulmonary edema is supplementary oxygen and ventilatory support with a high PEEP. The use of steroids, diuretics, and bronchodilators is also beneficial. As rapid reexpansion of a collapsed lung or a sudden increase in the negative intrapleural pressure can lead to fluid transudation across the capillaries and alveolar membranes, inhibitors of neutrophil elastase may serve as a rational treatment for patients with RPE. Trachiotis et al. [25] recommended that the lateral decubitus position was beneficial because it facilitated the recovery of insulted lungs from reduced perfusion and interstitial edema. Differential lung ventilation was recently advocated as a useful treatment for RPE [26]. Tung et al. [27] reported a case of severe RPE that developed bilaterally, in which they successfully cured the patient using extracorporeal membrane oxygenation. The reported mortality rate for RPE is very high. Mahfood et al. [17] reported that 11 of 47 patients with RPE died: the mortality rate is estimated as higher than 20%. It is likely that the early introduction of ventilatory support with high PEEP and the timely use of steroids and a neutrophil elastase inhibitor were beneficial for the complete recovery of the patient in the present case. 

In conclusion, we have described a case of RPE following an uneventful LADG for early gastric cancer. Single-lung ventilation may be a risk factor for RPE during laparoscopic surgery with pneumoperitoneum. Surgeons and anesthesiologists involved in laparoscopic surgery should be aware of the risk for this life-threatening disease. 

## Figures and Tables

**Table 1:** Perioperative ventilatory support information and arterial blood gas analysis results.

	Start of anesthesia	During surgery*	Bedroom	Reintubation	1 POD	3 POD	7 POD
Respirator mode	SIMV	SIMV		^†^SIMV (VC) + PS	^†^SIMV (VC) + PS	^†^Spont/PEEP + PS	
Tidal volume	400 mL	400 mL		450 mL	450 mL	450 mL	
Frequency	20 times	20 times	None	20 times	20 times	20 times	None
PS	0 mmHg	0 mmHg		10 mmHg	12 mmHg	10 mmHg	
PEEP	0 mmHg	0 mmHg		10 mmHg	10 mmHg	5 mmHg	

BGA							
FiO_2_	0.4	0.5	10 L mask	1.0	0.65	0.4	Room air
pH	7.414	7.384	7.237	7.328	7.338	7.397	7.420
*p*O_2_ (mmHg)	178.5	86.2	56.2	158.6	137.6	74.4	88.2
*p*CO_2_ (mmHg)	41.6	41.4	63.9	39.4	48.5	53.4	42.3
B.E. (mmol/L)	1.3	0.8	−2.4	−2.0	−0.9	6.4	1.0

SIMV: synchronized intermittent mandatory ventilation; VC: volume control; PS: pressure support; Spont: spontaneous respiration; PEEP: positive end-expiratory pressure; BE: base excess; POD: postoperative day; BGA: blood gas analysis; *During surgery: 229 minutes after the initiation of surgery. ^†^Puritan Bennett 840 Ventilator System.

**Table 2:** Reported cases of pulmonary edema following laparoscopic surgery.

Case	Year [Ref.]	Age	Sex	Comorbidity	Disease	Laparoscopic procedure	Position	Single-lung ventilation	Operation time	Infusion	Urinary output	Pulmonary edema
1	1995 [5]*	32 y	F	None	Sterility	Diagnostic laparotomy	Trendelenburg	Present	80 min	2000 mL	ND	Unilateral
2	2000 [6]	31 y	F	Obesity, pregnancy	Cushing's synd.	Adrenalectomy	Lateral	None	150 min	2150 mL	1100 mL	Unilateral
3	2005 [7]	23 y	F	Obesity	Obesity	Bariatric surgery	ND	Present	140 min	2400 mL	120 mL	Unilateral
4	2007 [8]	32 y	M	None	Donor	Nephrectomy	Lateral	None	300 min	7700 mL	1550 mL	Unilateral
5	2007 [8]	44 y	M	None	Donor	Nephrectomy	Lateral	None	330 min	8000 mL	2750 mL	Unilateral
6	2010 [9]*	45 y	M	None	Cecal cancer	Ileocecal resection	ND	None	182 min	3460 mL	1330 mL	Bilateral
7	2010 [10]*	73 y	M	HT, DM, angina	Cholecystitis	Cholecystectomy	ND	None	128 min	2150 mL	290 mL	Bilateral
8	2010 [11]	25 y	F	None	Ectopic pregnancy	ND	Trendelenburg	None	50 min	1750 mL	ND	Bilateral
9	2010 [12]	63 y	M	None	Prostate cancer	Prostatectomy	Trendelenburg	None	256 min	2500 mL	800 mL	Bilateral
10	2011^†^	57 y	F	None	Gastric cancer	Distal gastrectomy	Head-up tilt	Present	309 min	2150 mL	290 mL	Bilateral

*Reported in Japanese with English abstract; ^†^our case; Ref.: reference number; ND: not described; HT: hypertension; DM: diabetes mellitus; Synd.: syndrome.
